# Effect of Alpha-1 Antitrypsin Deficiency on Zinc Homeostasis Gene Regulation and Interaction with Endoplasmic Reticulum Stress Response-Associated Genes

**DOI:** 10.3390/nu17111913

**Published:** 2025-06-02

**Authors:** Juan P. Liuzzi, Samantha Gonzales, Manuel A. Barbieri, Rebecca Vidal, Changwon Yoo

**Affiliations:** 1 Department of Dietetics and Nutrition, Robert Stempel College of Public Health and Social Work, Florida International University, Miami, FL 33199, USA; rvida034@fiu.edu; 2 Department of Biostatistics, Robert Stempel College of Public Health and Social Work, Florida International University, Miami, FL 33199, USA; samagonz@fiu.edu; 3 Department of Biological Sciences, College of Arts, Sciences & Education, Florida International University, Miami, FL 33199, USA; barbieri@fiu.edu

**Keywords:** zinc, endoplasmic reticulum, liver, alpha-1 antitrypsin deficiency, Bayesian networks, machine learning, artificial intelligence

## Abstract

Background: Alpha-1 antitrypsin deficiency (AATD) is a genetic disorder caused by mutations in the *SERPINA1* gene, leading to reduced levels or impaired alpha-1 antitrypsin (AAT) function. This condition predominantly affects the lungs and liver. The Z allele, a specific mutation in the *SERPINA1* gene, is the most severe form and results in the production of misfolded AAT proteins. The misfolded proteins accumulate in the endoplasmic reticulum (ER) of liver cells, triggering ER stress and activating the unfolded protein response (UPR), a cellular mechanism designed to restore ER homeostasis. Currently, there is limited knowledge regarding specific nutritional recommendations for patients with AATD. The liver is essential for the regulation of zinc homeostasis, with zinc widely recognized for its hepatoprotective properties. However, the effects of AATD on zinc metabolism remain poorly understood. Similarly, the potential benefits of zinc supplementation for individuals with AATD have not been thoroughly investigated. Objective: This study explored the relationship between AATD and zinc metabolism through a combination of in vitro experiments and computational analysis. Results: The expression of the mutant Z variant of ATT (ATZ) in cultured mouse hepatocytes was associated with decreased labile zinc levels in cells and dysregulation of zinc homeostasis genes. Analysis of two data series from the Gene Expression Omnibus (GEO) revealed that mice expressing ATZ (PiZ mice), a murine model of AATD, exhibited significant differences in mRNA levels related to zinc homeostasis and UPR when compared to wildtype mice. Bayesian network analysis of GEO data uncovered novel gene-to-gene interactions among zinc transporters, as well as between zinc homeostasis, UPR, and other associated genes. Conclusions: The findings provide valuable insights into the role of zinc homeostasis genes in UPR processes linked to AATD.

## 1. Introduction

Alpha-1 antitrypsin deficiency (AATD) is an autosomal recessive disorder caused by mutations in the *SERPINA1* gene (AAT). AATD can lead to damage in the lungs and liver, resulting in conditions such as emphysema and liver disease [1]. *SERPINA1* is primarily synthesized in the liver and released into plasma, where it serves as a circulating protease inhibitor. The Z and S alleles are the most common mutations associated with AATD [1]. The Z allele results in the substitution of glutamate for lysine at amino acid position 342 (p. *Glu324Lys)*, which alters the peptide folding [1] and affects its secretion, causing severe deficiency in blood. The mutant *SERPINA1* (AATZ) molecules undergo polymerization and aggregate within the endoplasmic reticulum (ER) of hepatocytes [2]. This leads to ER stress and the formation of large intrahepatocyte globules, necrosis, fibrosis, and apoptosis [1].

Currently, there is a limited body of formal research examining the necessity of specific nutritional recommendations for patients with AATD [3]. Additionally, studies investigating the effects of AATD on nutrient metabolism remain scarce. Ongoing research is shedding light on the role of iron in AATD pathology. Schaefer et al. [4] reported that patients with AATD, specifically those with the Z mutation, exhibit hepatic iron overload alongside reduced hepcidin levels compared to control patients. Moreover, they demonstrated that AAT reduced the cleavage of the hepcidin-inducing bone morphogenetic protein co-receptor HJV via inhibition of the membrane-bound serine protease MT-2 in cultured cells, which can result in increased hepcidin expression in vivo [4].

Furthermore, other studies have shown that AATD enhances the expressivity of hemochromatosis [5,6].

The liver plays a critical role in zinc metabolism [7]. Any dysfunction in the liver, such as in conditions like AATD, may disrupt zinc metabolism and contribute to imbalances in the body. Nonetheless, the impact of AATD on zinc metabolism and status remains undocumented. There is general agreement that zinc functions as a hepatoprotective nutrient via its role in mitigating inflammation, fibrosis, steatosis, and apoptosis and stimulating autophagy [8,9,10,11,12,13]. However, it is uncertain whether zinc supplementation offers any benefits to patients with AATD. Additionally, it is unclear whether poor zinc intake aggravates AATD-driven pathology.

Interestingly, reports have indicated a connection between ER stress, early secretory pathway, and zinc [14]. Approximately one-third of cellular proteins are targeted to the ER, where they undergo folding and post-translational modifications, such as glycosylation [15]. Properly folded proteins are transported to the Golgi apparatus and other destinations, while misfolded ones may be targeted for degradation through the ER-associated degradation (ERAD) pathway. A significant proportion of the secretome requires zinc for its structural and catalytic functions. Additionally, chaperones require zinc for regulation and proper function [16]. Strengthening the idea that zinc is essential for ER function, a study revealed that low zinc intake intensified ER stress induced by tunicamycin in the liver of mice [17].

Furthermore, loss-of-function studies of zinc transporters have highlighted the critical role of zinc in ER function. There are two families of zinc transporters in vertebrates [18]. The transporters from the *Slc39a* gene family, in most cases, increase intracellular zinc levels by importing zinc into the cytoplasm from outside the cell or intracellular storage sites. With some exceptions, most *Slc30a* transporters help reduce intracellular zinc by exporting it out of the cell or sequestering it into specific organelles. *Slc30a5* has been identified at both the Golgi apparatus and the endoplasmic reticulum (ER) [19], while *Slc30a6* and *Slc30a7* are localized exclusively at the Golgi apparatus. Notably, *Slc30a5* and *Slc30a6* can form functional heterodimers; however, the specific localization of these heterodimers remains poorly characterized [14,20,21,22].

These transporters seem to be critical for zinc transport in early secretory pathway and cells lacking these transporters exhibit increased ER stress [23]. *Slc39a7* has been localized to the endoplasmic reticulum (ER), where it is believed to play a role in facilitating zinc release from this compartment [24]. Supporting this proposed function in the ER’s zinc transport, studies have demonstrated that the abrogation of this gene leads to zinc accumulation within the ER, resulting in ER stress in mesenchymal stem cells [25]. Additionally, Kim et al. [26] demonstrated that *Slc39a14* plays a vital role in mitigating excessive ER stress responses. Furthermore, their findings revealed that the expression of this transporter is upregulated by ER stress or unfolded protein response (UPR) transcription factors, specifically Atf4 and Atf6α.

Given the strong association between AATD and ER stress, it is plausible that AATD contributes to the dysregulation of zinc homeostasis-related genes through ER stress-mediated mechanisms.

In this study, we aimed to investigate the impact of Z mutation on zinc metabolism and its broader implications for genetic regulation. Specifically, we focused on the co-regulation of genes involved in zinc homeostasis, UPR, and other pathways, aiming to uncover potential interactions and underlying mechanisms. To achieve this, we utilized Bayesian network (BN) analysis, which is particularly well-suited for studying the co-regulation of many genes because it can handle complex, high-dimensional datasets while incorporating uncertainty.

## 2. Materials and Methods

### 2.1. Cell Culture

Mouse hepatocytes (Hepa-1-6) with constitutive expression of the human *SERPINA1* (AAT) wildtype allele (M) (WT) and the mutant Z allele (ATZ) were kindly donated by Dr. Jeffery Tekman [27]. The cells expressing the mutant allele exhibit increased retention of mutant Z protein in the ER and abnormal basal autophagy [27]. Cells were cultured in DMEN medium containing 10% FBS and antibiotic-antimycotic. Cell culture procedures were approved by the Institutional Biosafety Committee of Florida International University.

### 2.2. Labile Zinc and Cell Viability Measurement

To test the effect of zinc depletion, adequacy, and excess, cells were seeded in 96-well plates (20 × 10^4^ cells per well) and treated 24 h later with either the zinc chelator TPEN (5 μM) (N,N,N′,N′-Tetrakis(2-pyridylmethyl)ethylenediamine)(Sigma-Aldrich, St. Louis, MO, USA), vehicle alone (DMSO), or vehicle plus zinc (40 μM) (ZnSO_4_) for 24 h. Thereafter, cells were incubated with the cell-permeable zinc-fluorophore NBD-TPEA (Sigma-Aldrich, St. Louis, MO, USA) as described before [13]. Labile zinc was quantified using a Biotek FLx800 plate reader (excitation 485 nm/emission 528 nm). Values were normalized to Hoechst fluorescence. Cell viability was assessed by measuring lactate dehydrogenase (LDH) leakage using a cytotoxicity LDH assay kit (Dojindo, Rockville, MD, USA).

### 2.3. mRNA Quantitation by RT-PCR

Total RNA was isolated from cells utilizing the RNeasy plus kitRNA (Qiagen, Hilden, Germany), which allows genomic DNA removal. Quantitative PCR (qPCR) was performed using ABScript II One Step SYBR Green RT-qPCR kit (ABclonal, Woburn, MA, USA). The master mix qPath (Thermo Fisher, Waltham, MA, USA) was also employed for the measurement of mRNA expression of *Slc39a4* and *Mt1*.

The following set of primers were utilized for qPCR using SYBR Green: Mt2: 5′-GCC TGC AAA TGC AAA CAA TGC-3′ and 5′-AGC TGC ACT TGT CGG AAG C-3′; *Slc39a8*: 5′-ATC TGC CCC GCG ATC TTA C-3′ and 5′-CCC CAG ACT TCG GAA AGA CT-3′; r18s: 5′-GTAACCCGTTGAACCCCATT-3′ and 5′-CCATCCAATCGGTAGTAGCG-3′. *Slc39a14*: predesigned primers Quantitec (Qiagen). Mouse *Slc39a4* and *Mt1* mRNA expression was measured using TaqPath 1-step RT-qPCR mix CG (Thermo Fisher, Waltham, MA, USA). The primers used for *Slc39a4*: 5”-CTCTGCAGCTGGCACCAA-3′ and 5′-CACCAAGTCTGAACGAGAGCTTT-3′ and the FRET probe (5′ FAM; 3” BHQ1) sequence was: 5′-CAATCTCCGACAGTCCAAACAGACCCAT-3′ (R) as previously reported [28]. Mouse *Mt1* mRNA expression was measured using the predesigned primer and probe Mm496660.g1 (Thermo Fisher, Waltham, MA, USA). Values were normalized by r18s.

### 2.4. Western Blot Analysis

Insoluble fractions of cells were obtained by lysing cells in lysing buffer (50 mM Tris and 1% Triton X-100) containing protease inhibitors and 5mM EDTA (Halt protease inhibitor cocktail; Pierce, Rockford, IL, USA). To isolate the insoluble fractions, lysates were centrifuged at 16,000× *g* for 15 min. To solubilize the aggregates, lysates were briefly sonicated. Cellular protein content was determined with a micro-BCA protein assay kit (Pierce, Rockford, IL, USA). Aliquots of homogenates were diluted with 5× Laemmli sample buffer and resolved on 4–12% polyacrylamide gels (Genscript). Proteins were transferred onto nitrocellulose membranes using the eBlot^®^ protein transfer system (Genscript, Piscataway, NJ, USA). The detection of AAT was achieved using the monoclonal antibody CPTC-SERPINA1-1 (DSHB Hybridoma, Iowa, IA, USA). β-Tubulin was used as a loading control (Cat #5346; Cell signaling). Densitometry values were normalized to total protein (ponceau S staining).

### 2.5. GEO Data Analysis

We downloaded raw count data from the GEO dataset GSE141593, which contains RNA-seq data from livers from five 6-week-old male PiZ mice (expressing human Z mutant allele), a murine model of AATD, and five C57BL/6 (wildtype) mice [29]. We used the GEOQuery package [30] in R (4.4.3). Gene expression counts were filtered for low expression by removing genes whose expression counts were less than 10 in five or more samples. To select genes for input to the BN analysis, differential expression analysis was performed on the raw count data using R package DESeq2 [31], and genes with an ‘apleglm’ shrunken log2 fold change greater than 2 (absolute lg fold change) as well as zinc-related genes of interest were selected, for a total of 215 genes. Counts for the entire dataset were first normalized using DESeq2′s Ratio of Medians method, standardized to Z scores, and finally discretized into 3 categories: low expression (z < −1, denoted as 1), no change in expression (−1 ≤ z ≤ 1, denoted as 0), and high expression (z > 1, denoted as 2). The transcriptomic data of the GEO dataset GSE93115 [32] was employed only for gene expression comparison with GSE141593. GSE93115 contained microarray data (Affymetrix Mouse 430A 2.0 array). This dataset contains data from liver samples from three 6-week-old male PiZ mice and three C57BL/6 (wildtype) mice. The log2 fold change and *p* values for comparison between PiZ mice and wildtype were obtained by using GEO2R.

### 2.6. Bayesian Network Analysis

**Figure 1:**
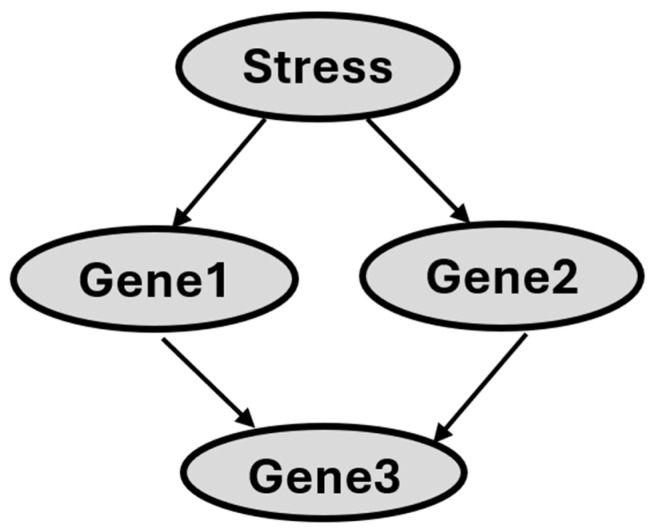
Diagram illustrating hypothetical gene–gene interactions in response to stress.

A BN is a directed acyclic graph in which each arc is interpreted as a direct influence between a parent node and a child node, relative to the other nodes in the network [33]. Figure 1 shows the structure of a BN that illustrates hypothetical gene–gene interactions in response to stress. The nodes in Figure 1 (depicted as ovals) represent random variables, and each node is associated with a set of possible states. As an example, the ‘Stress’ node can take on the states ‘Yes’ or ‘No’ to indicate the presence or absence of stress exposure in the sample. Likewise, the ‘Gene1′ node can have states such as ‘High’, ‘no change’, or ‘low’, representing the gene’s expression level. Figure 1′s arcs show direct variable relationships. Arc direction indicates parent–child status: for example, ‘Stress’ is a direct parent of Gene1 and Gene2, and Gene3 is a direct child of Gene2. A set of probabilities parameterizes the structure of BN. In general, for each variable, there is a conditional probability of that variable given the states of its parents. Thus, the probability associated with a set of variables in Gene3 is P(Gene3, Gene1, Gene2). That is, we give the probability distribution over the values of a set of variables in Gene3 conditioned on each of the possible values of a set of variables in Gene1 and Gene2. For variables that have no parents in the network, a prior probability is specified. The Markov condition [33] specifies the conditional independence relationships that are represented by a BN network: Let X and Y be variables. Suppose that Y is neither a direct nor an indirect child of X. Then X is independent of Y, conditioned on any state of the direct parent of X.

BN analysis was conducted using the scoring metric Bayesian Dirichlet equivalence (BDe) [34] and BANJO (BN Inference with Java Objects) (Version 2.0) [35] that iteratively modified the network structure to maximize the BDe score was used to uncover gene–gene interactions from PiZ and wildtype datasets, helping to elucidate gene–gene interactions in PiZ mutation. Our approach included a uniform BDe prior, meaning we assumed all joint probability distributions that fit the network structure were equally likely. First, three independent searches were carried out with Banjo’s simulated annealing algorithm, each running for 2 h. The highest-performing structure from these searches (based on normalized log likelihood scores) was used as the starting point for the next round of three independent searches, now set to run for 4 h each. The best structure from this second set was then compared to the best structure from the initial round by assessing the normalized scores. Our approach included a uniform BDe prior, meaning we assumed all joint probability distributions that fit the network structure were equally likely. If the improvement was no greater than 15%, the search was concluded. Otherwise, the best structure from this recent round became the initial structure for a subsequent search, which had double the runtime. A total of three replicates were conducted for each of four search durations: 1 h, 2 h, 4 h, and 8 h, leading to a cumulative computing time of 45 h.

A Markov Blanket (MB) in BN refers to the set of nodes that shield a particular node from the rest of the network, meaning the node is conditionally independent of all other nodes given its MB. The first-degree MB of a node includes its parents, children, and the parents of its children. The second-degree MB of a node is composed of its first-degree MB along with the first-degree MB of each member within its first-degree MB.

Following the BDe search method described earlier, we first searched for the optimal BN using the 215 genes identified in the GEO data analysis section. From this initial optimal BN, we then focused on the second-degree MB of PiZ and incorporated genes of interest to arrive at a set of 92 genes. Finally, we applied the BDe search method again to determine the final best BN for these 92 genes. In this final best BN, we focused specifically on genes within the first-degree MB of PiZ in a BN, denoted as MB(PiZ). We then extended our analysis to genes in the second-degree MB of PiZ, denoted as MB(MB(PiZ)).

To investigate potential gene interactions under PiZ and wildtype conditions, we performed an in-depth analysis of all possible gene expression state combinations for the eight genes selected from MB(PiZ). These eight genes were identified based on their expression profiles and existing background knowledge. We determined the minimal set of genes necessary and identified the most probable two- and three-gene combinations for each condition. This method enabled us to uncover potentially novel gene–gene interactions.

### 2.7. Statistical Analysis

Data are presented as means ± standard error (SE). Two-way ANOVA or one-way ANOVA was used followed by the post hoc test Student–Newman–Keuls. Student’s *t*-test was also utilized. Statistical significance was set at *p* < 0.05.

## 3. Results

### 3.1. Expression of AAT

**Figure 2:**
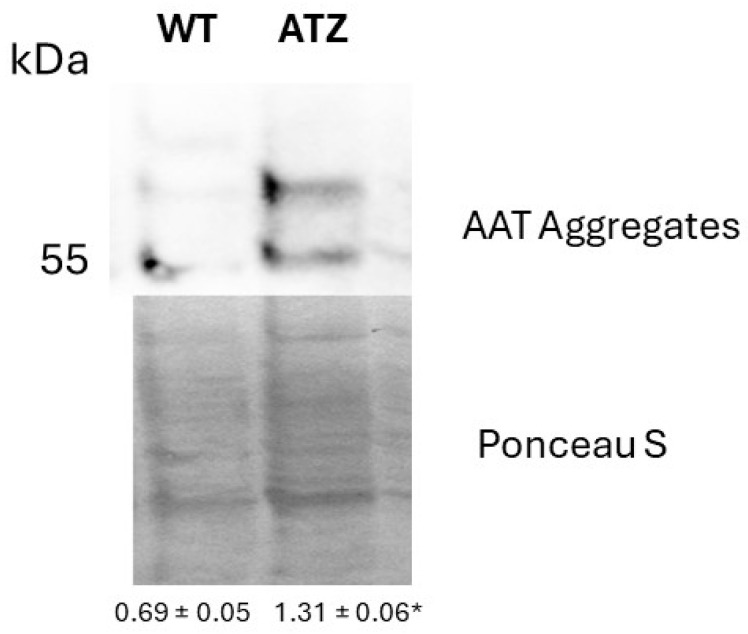
Representative Western blot showing AAT expression in Hepa-1-6 expressing either human wildtype (WT) or Z mutant (ATZ) AAT. Densitometry values of 55 kDa band. Values are averages ± 1 S.D of three independent experiments. * Significantly different according to Student’s *t*-test (*p* < 0.05).

Hepatocytes expressing ATZ showed elevated levels of ATT (~55 kDa) in the insoluble fractions of protein lysates, as compared to wildtype cells (WT), suggesting the accumulation of ATT aggregates (Figure 2).

### 3.2. Labile Zinc

**Figure 3:**
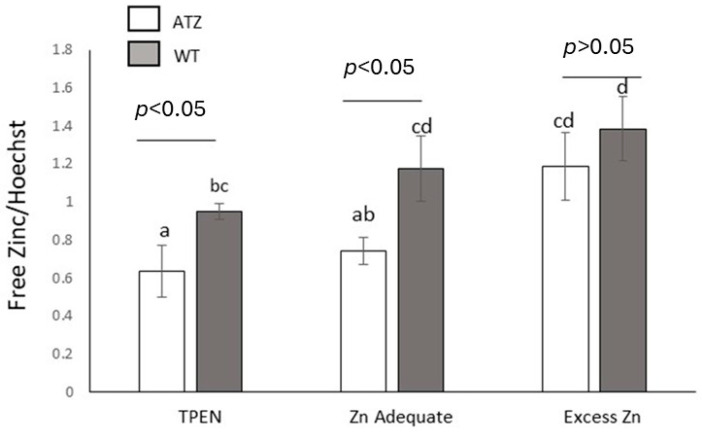
Effect of human ATT (wildtype (WT) or mutant Z (ATZ)) expression on labile zinc. Cells were treated with TPEN (5 μM), Zn Adequate (vehicle alone (DMSO)), or Excess Zn (vehicle plus 40 µM of zinc) for 24 h. Different superscript letters indicate statistical significance *p* < 0.05. Values are averages ± 1 S.D of three independent experiments. Two-way ANOVA interaction: cell type vs. zinc levels (*p* > 0.05).

Hepatocytes expressing ATZ exhibited lower labile zinc levels as compared to wildtype cells (WT) (*p* < 0.05), under both zinc deficiency (TPEN) and adequacy conditions (Figure 3). However, no significant difference was observed between these cells when they were cultured in medium with elevated zinc content. The treatments did not affect the viability of cells, as indicated by LDH leakage measurement (Figure S1).

### 3.3. Expression of Zinc Transporters and Mt1 and Mt2 in Hepatocytes

**Figure 4:**
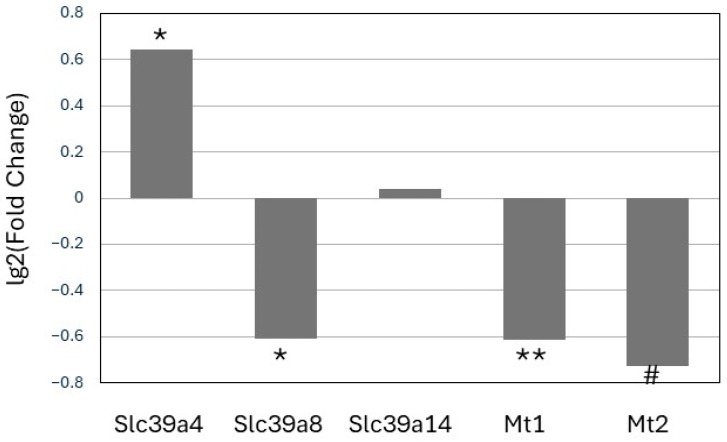
Differential expression of zinc transporters and *Mt1* and *Mt2* in Hepa 1-6 cells expressing either wildtype (WT) or mutant Z (ATZ) AAT. Values are Lg2 of fold change (ATZ/WT). Values are averages of three independent experiments. * *p* < 0.05; ** *p* < 0.001 Student’s *t*-test (two tails). # *p* < 0.05 Student’s *t*-test (one tail).

The expression of the zinc transporter *Slc39a4* mRNA was significantly increased (*p* < 0.05) in cells expressing ATZ as compared with wildtype cells (Figure 4). On the other hand, the expression of ATZ was associated with decreased mRNA expression of the zinc transporter *Slc39a8* as well as lower *Mt1* and *Mt2* expression. Lastly, the mRNA levels of *Slc39a14* were not affected by the mutation.

### 3.4. Gene Expression of Genes Involved in Zinc Homeostasis and UPR in Liver Samples of PiZ and Wildtype Mice (GEO)

The expression of zinc transporters, metallothioneins, and the transcription factor MTF1 (Metal Regulatory Transcription Factor 1) was analyzed in two independent GEO data series (NCBI) using PiZ mice-a murine AATD model-and wildtype mice of the same age and gender (6-week-old male). *Mt1* and *Mt2* mRNA levels were consistently upregulated in both studies. The mRNA expression of six zinc transporters was significantly affected by the expression of the human Z allele (PiZ mice) in at least one study. Interestingly, we observed that the expression of Slc39a7 mRNA was higher in PiZ mice, as compared to wildtype in both studies. However, the expression of *Slc39a1*, *Slc39a3*, *Slc39a4*, and *Slc39a11* was increased only in one study. On the other hand, the expression of *Slc39a8* and *Slc30a9* was significantly lower in PiZ mice in one study (Table 1). As for the expression of UPR genes (Table 2), the expression of the UPR-associated genes *Atf3*, *Atf4*, *Derl3*, *Trib3*, *Ddit3*, *Ddit4, Niban1* was significantly upregulated in PiZ mice in both series. However, the expression of *Sdf2l1* and *Bhlha15* was significantly upregulated by the mutation in one study.

### 3.5. Bayesian Networks Analysis

Utilizing the RNA-seq data from GSE141593 (wildtype vs PiZ mice n = 5 each group), we developed a network focused on zinc transporters, *Mt1*, *Mt2*, and *MTF1* (zinc homeostasis genes) as well as on UPR-associated genes. The cutoff point for gene inclusion in the analysis was lg2fc 2 (absolute value (plus and minus)). The zinc homeostasis and UPR-associated genes (included in Table 1 and Table 2) that did not reach lg2fc were added to the analysis as genes of interest. This probabilistic type of analysis does not fully establish causality or direction. However, it determines co-regulation or inhibition and provides potential direction.

**Figure 5:**
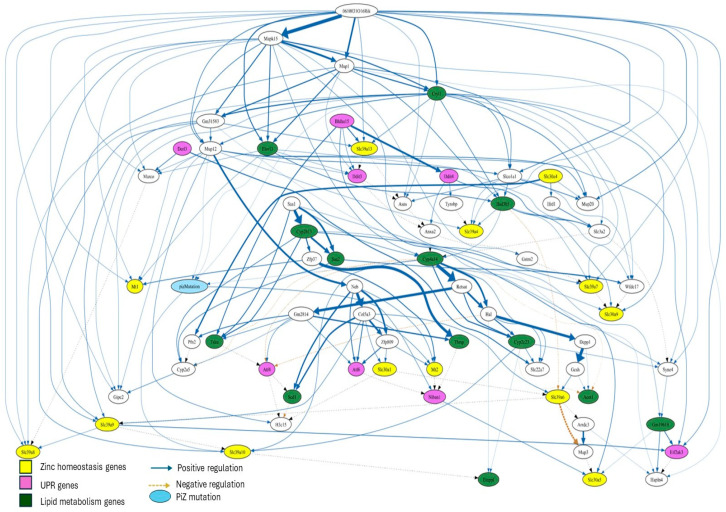
Second-degree Markov blanket of PiZ. The significance of connection between genes is proportional to the thickness of the lines. In addition, dotted lines indicate inhibition.

**Figure 6:**
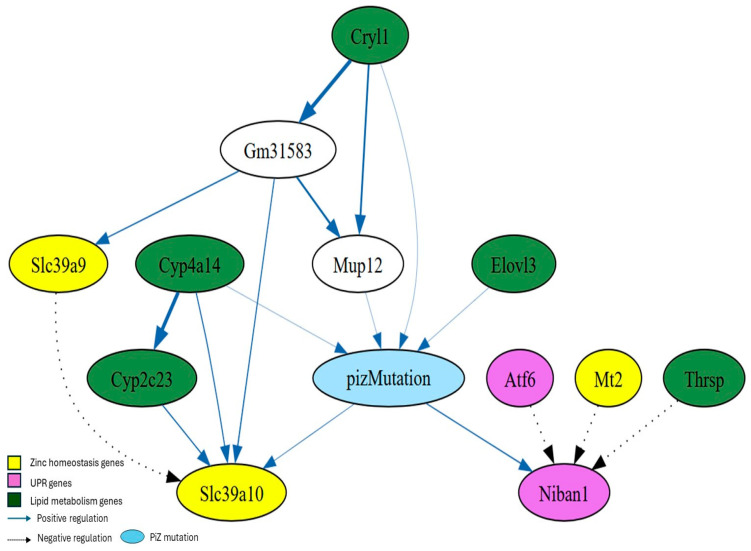
First-degree Markov Blanket of PiZ. The significance of connection between genes is proportional to the thickness of the lines. In addition, dotted lines indicate inhibition.

The BN structure that best fits the data revealed a second-degree MB of PiZ, defined as MB(MB(PiZ)) earlier, consisting of 68 genes (Figure 5), while the first-degree MB of PiZ comprised 12 genes (Figure 6).

The first-degree MB of PiZ shows the genes (nodes) that are in close association with the PiZ mutation. Specifically, the MB of PiZ consists of PiZ’s parents, children, and the co-parents (nodes that share a child with the PiZ mutation) (Figure 6). Genes associated with zinc homeostasis (*Mt2*, *Slc39a9*, and *Slc39a10*) [18], UPR (*Atf6* and *Niban1*) [36], and lipid metabolism (*Elovl3*, *Thrsp*, *Cyp2c23*, *Cyp4a14*, and *Cryl1*) [37,38,39,40,41] were identified within the first-degree MB. *Mup12* and *Gm31583* were part of the first-degree MB; however, their biological roles are not well defined. *Mup12* or Major Urinary Protein 12, is a protein-coding gene found in mice. This gene belongs to the lipocalin family, which is involved in transporting small hydrophobic molecules such as pheromones [42].

The first-degree MB of PiZ reveals that the PiZ mutation is directly linked to *Slc39a10*, which, in turn, is associated with *Slc39a9* through an inhibitory interaction. *Mt2*, *Atf6* and *Thrsp* are not directly connected to the PiZ mutation but have an inhibitory interaction with *Niban1* (Figure 6).

The combinations of 2 and 3 genes “status” with maximum probabilities associated with the PiZ mutation within the first-degree MB of PiZ were determined (Table 3). The expression of the UPR-associated gene Niban1 was found to be “high” when in combination with the genes *Elovl3* and *Mt2* or *Cyp4a14* and *Thrsp* “under no change” status.

The second-degree MB of PiZ extends beyond the first-degree MB of PiZ by including nodes that are two steps away from the target node (PiZ). This MB contains a total of thirteen genes associated with zinc homeostasis and lipid metabolism. However, a total of eight UPR-associated genes were identified within this network (Figure 5). The network shows significant connections between some of these three types of genes as well as with genes involved in other biological processes. The diagram suggests that the expression of *Slc39a13* is positively regulated by the UPR-associated genes *Bhlha15* and *Ddit3*. A similar interaction was found between *Slc30a5*, *Bhlha15*, and *Niban1*. Additionally, *Slc39a9* is connected to *Slc39a6*, *Slc39a8*, *Slc39a10*, and the UPR-associated gene *Eif2ak3*.

*Slc39a7* was found to be connected to *Slc30a9*, *Mapk15*, the long non-coding RNA (lncRNA) 0610031O16Rik, *Gstm2*, *Cryl1*, and *Hsd3b5*.

Lastly, 0610031O16Rik was at the top of the network and exhibited connections (as positive regulator) with multiple genes, including *Mapk15*, *Mt1*, *Slc39a7*, *Slc39a8*, and *Eif2ak3*. The analysis indicated a connection with strong significance (thicker line) between 0610031O16Rik and *Mapk15*.

### 3.6. GeNIe Simulations

We carried out simulations utilizing GeNIe (Bayes Fusion LLC, Pittsburgh, PA, USA) in which we artificially modified the expression of *Slc39a7* as well as the mutation conditions: PiZ and wildtype (Figure S2). We observed that, under PiZ mutation (simulated 100%) (Figure S2B), and simulated high expression (100%) of *Slc39a7*, there was an important increase in the expression of the UPR-associated genes *Atf4*, *Ddit3*, *Bhha15*, *Eif2ak3*, and *Niban1*, and the zinc homeostasis gene *Slc39a13*. However, the expression of *Slc30a5* and *Slc30a9* decreased. On the other hand, when the condition was set to “wildtype” 100% and “high *Slc39a7*” (Figure S2C), the expression of most UPR-associated genes, *Slc39a13*, and *Slc30a5* became mostly “no change” or low in the case of *Eif2ak3*. Interestingly, the expression of *Slc39a9* decreased while *Slc39a8* and *Slc39a10* expression increased. However, the expression of *Slc30a9* remained low.

## 4. Discussion

To our knowledge, this is the first study that investigates the effect of AATD on zinc metabolism. Additionally, this is the first study to use BN analysis to evaluate the co-regulatory relationship between zinc homeostasis, UPR-associated genes, and other genes using a murine model of AATD. The studies with hepatocytes indicated that expression of the mutant Z version of AAT had a detrimental effect on labile zinc in cells under both zinc deficiency and zinc adequate conditions. This suggests that the Z mutation may be detrimental to hepatocytes’ zinc status. Interestingly, the mutation did not significantly affect labile zinc in cells exposed to excess zinc. Since the treatments did not affect cell viability, it is unlikely that the observed changes in labile zinc resulted from decreased viability in mutant cells.

Since the intracellular distribution of labile zinc was not examined, it is unknown whether the mutation affected the intracellular localization of labile zinc. Furthermore, it is uncertain whether the total zinc content in cells was affected by the mutation. Moreover, this study did not assess the impact of the mutation on zinc transport kinetics.

It is important to note that these transgenic cell lines may yield artifactual results due to insertional effects.

Notably, the expression of the Z allele was also associated with the dysregulation of several zinc transporters, as well as Mt1 and Mt2 in cultured hepatocytes and in liver samples from mice (GEO data series). However, it is essential to note that the dysregulation of these genes was not entirely consistent, and some results showed divergence.

It is not clear why the “in vitro study” and the studies in mice produced different results. Hormonal and immune factors, as well as systemic interactions, may play a role. Metallothioneins are known to be upregulated by glucocorticoids and by inflammation [43,44], which could influence their expression in the liver in mice.

Additionally, it is important to mention that the wildtype used in the “in vitro” hepatocyte study overexpresses the human M variant (considered wildtype) of AAT, whereas the wildtype mice, serving as controls for the PiZ mice, do not express human AAT. Lastly, the cells used in the in vitro study are mouse hepatoma cells rather than primary cells, which may also contribute to differences between studies conducted in mice and in cells.

On the other hand, the techniques employed to obtain the transcriptomic data of the two data series differ, which may have contributed to the differences observed between these two studies. GSE93115 contains data originating from cDNA microarray analysis, and GSE141593 data originated from RNA-seq analysis.

The BN analysis helped identify novel interactions between zinc transporters, UPR-associated genes, other genes, and the PiZ mutation in the liver of mice.

The first-degree MB of PiZ revealed a close relationship between the PiZ mutation and *Slc39a10*, *Slc39a9*, and *Mt2*. Alterations in the expression of these genes could influence zinc levels within specific cellular compartments, triggering signals that initiate intracellular responses.

*Slc39a9* has been identified at the trans-Golgi network, plasma membrane, mitochondria, and nucleus [45,46]. This transporter is thought to contribute to zinc homeostasis within the secretory pathway [45]. In contrast, *Slc39a10* is localized at the plasma membrane of hepatocytes, and its expression is negatively regulated by zinc levels [47]. The first-degree MB of PiZ also shows an inhibitory interaction between *Slc39a9* and *Slc39a10* (Figure 6). Perhaps, *Slc39a9*′s zinc transport activity has a negative impact on the expression of *Slc39a10*.

The second-degree MB of PiZ provided additional information regarding the interactions among genes. *Slc39a7* is located at the ER, and its upregulation is expected to decrease zinc content in the ER, which increases ER stress. Noteworthy, *Slc39a7* was found to be consistently upregulated in both mice studies (Table 1).

**Figure 7:**
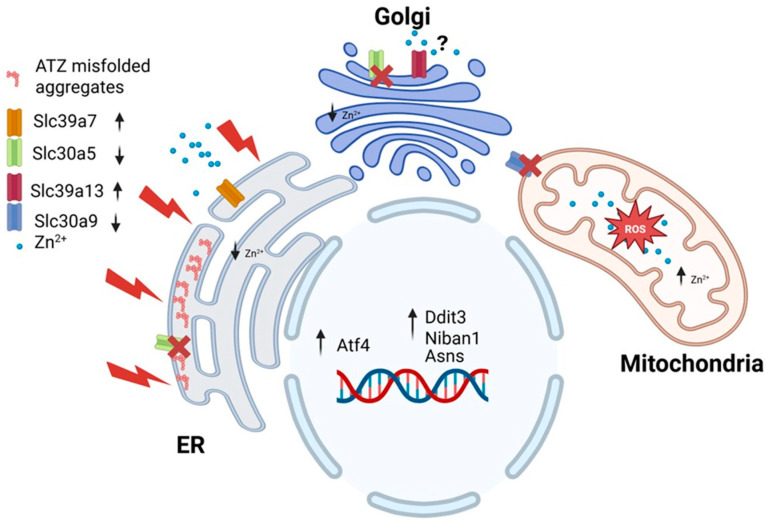
Diagram representing GeNIe simulation when *Slc39a7* is set to high expression under 100% PiZ mutation. Zinc content in the ER and Golgi apparatus is decreased, which enhances ER stress. Downregulation of *Slc30a9* at mitochondria may lead to mitochondrial stress. Created with Biorender (Biorender.com).

The GenNIE simulations allowed us to investigate the effect of artificial upregulation/downregulation of a particular gene on the expression of other genes. Interestingly, when *Slc39a7* expression was set to high 100% (Figure S2B), there was an increased expression of key UPR-linked genes under PiZ but not under wildtype condition (Figure S2C). This suggests that *Slc39a7* upregulation could be part of the UPR response associated with the Z mutation (Figure 7). Supporting the notion that this gene is involved in the UPR), its expression was shown to be upregulated in 293T cells following exposure to the ER stress inducers tunicamycin and thapsigargin [48].

Interestingly, the expression of *Slc30a5* and *Slc30a9* appears to be inversely regulated with *Slc39a7*, suggesting a negative correlation in their regulatory dynamics (Figure S2B). However, only the expression of *Slc30a5* returned to mostly “no-change” under the “wild-type” condition (Figure S2C). This suggests that the coregulation between *Slc39a7* and *Slc30a9* appears to function independently of the PiZ mutation status.

Given the location of *Slc30a5*, the downregulation of this transporter is expected to decrease levels of zinc within the ER and Golgi apparatus (Figure 7). *Slc30a9* has been identified at the mitochondria, ER, and nucleus [48,49]. Deng et al. [49] highlighted the critical role of this transporter in facilitating zinc export from mitochondria in both *C. elegans* and human cells. Their study revealed that the absence of *Slc30a9* led to increased zinc accumulation within mitochondria, resulting in mitochondrial swelling and impaired metabolic function. This is not surprising because excess zinc can cause an increase of reactive oxygen species (ROS) and loss of mitochondrial membrane potential [50]. The role of *Slc30a9* at the ER remains unexplored. Nevertheless, data from GSE167299 revealed that the expression of this gene was significantly downregulated (*p* < 0.05) in the liver of mice following tunicamycin treatment [51]. This suggests that the downregulation of this gene may play a role in UPR.

The simulation also showed coregulation between *Slc39a7* and *Slc39a13* (Figure S2). However, this coregulation was enhanced by the presence of 100% PiZ mutation. *Slc39a13* has been identified at the Golgi apparatus [52]. Its upregulation is expected to facilitate zinc transport out of the Golgi, which, alongside the downregulation of *Slc30a5*, could lead to significant zinc depletion in the early secretory pathway (Figure 7). However, it is important to note the conflicting evidence regarding the role of *Slc39a13*. Jeong et al. [53] suggested that this transporter serves as an exporter of zinc from intracellular vesicles for utilization in the ER.

The coregulation of *Slc39a7* with *Slc30a5*, *Slc30a9*, and *Slc39a13* may indicate a crosstalk mechanism between the endoplasmic reticulum (ER) and mitochondria. During ER stress, the increased release of zinc from the ER and other compartments, coupled with a reduced export of this metal from mitochondria, could contribute to increased ROS production and apoptosis (Figure 7).

## 5. Conclusions

BNs offer distinct advantages over traditional statistics and bioinformatics in discovering gene-gene and gene-environment interactions. In this study, we employed this approach to gain insight into potential new regulatory mechanisms, particularly those involving zinc homeostasis and UPR-linked genes, without the use of laboratory experiments. Our study did not incorporate experimental validation of the BN predictions. Conducting validation studies would provide valuable insights into the predictive accuracy and reliability of BN.

A critical limitation of focusing solely on mRNA expression for BN analysis, particularly in the case of transcription factors and enzymes, is that it does not necessarily reflect their activity, which may depend on phosphorylation status or other post-translational modifications.

However, as AI becomes more sophisticated, it will be possible to incorporate more variables into the probabilistic prediction, allowing for the development of more realistic simulations of cell behavior.

## Figures and Tables

**Table 1:** Differential expression of zinc homeostasis genes in liver in wildtype and PiZ mice.

	GSE93115	GSE141593
*Mt1*	3.4 *	1.7 *
*Mt2*	3.5 *	3.6 **
*Mtf1*	−0.23	0.4
*Slc39a1*	−0.5 *	0.3
*Slc39a3*	0.6 *	−0.1
*Slc39a4*	0.3	1.8 **
*Slc39a6*	0.3	0.02
*Slc39a7*	0.6 *	1.3 **
*Slc39a8*	−0.6 *	−0.7
*Slc39a9*	0.5	−0.3
*Slc39a10*	NF	0.3
*Slc39a11*	0.3	1.4 **
*Sl39a13*	0.4	0.7
*Slc39a14*	0.3	−0.1
*Slc30a1*	−0.4	0.1
*Slc30a4*	−0.1	0.4
*Slc30a5*	−0.3	−0.02
*Slc30a6*	−0.4	0.1
*Slc30a7*	−0.02	0.3
*Slc30a9*	−0.5 *	−0.3

Data originated from GSE93115 (n = 3) [30] and GSE141593 (n = 5) [28]. Values are Lg2 of fold change of PiZ/wildtype mice. * *p* < 0.05; ** *p* < 0.001. NF = not found.

**Table 2:** Differential expression of genes associated with UPR in liver in wildtype and PiZ mice.

	GSE93115	GSE141593
*Atf3*	5.3 **	3.5 **
*Atf4*	0.9 *	0.8 **
*Atf6*	−0.5	0.1
*Ern1* (*IRE1*)	0.3	0.4
*Sdf2l1*	0.34	2.9 **
*Xbp1*	−0.9 *	−0.1
*Eif2ak3* (*Perk*)	0.3	0.6
*Derl3*	1.8 *	5.7 **
*Trib3*	3.8 **	3.0 **
*Ddit3* (*Chop*)	3.7 **	3.0 **
*Ddit4*	0.5 *	4.9 **
*Bhlha15* (*Mist1*)	0.3	6.7 **
*Niban1* (*Fam129a*)	1.05 *	2.3 **

Data originated from GSE93115 (n = 3) [30] and GSE141593 (n = 5) [27]. Values are Lg2 of fold change of PiZ/wildtype mice. * *p* < 0.05; ** *p* < 0.001.

**Table 3:** Conditional Probabilities of Nodes in First-degree Markov Blanket.

Condition	
PiZ	*Cyp4a14* [0], *Elovl3* [0]
PiZ	*Elovl3* [0], *Mt2* [0], *Niban1* [2]
PiZ	*Atf6* [0], *Cyp4a14* [0], *Elovl3* [0]
PiZ	*Cyp4a14* [0], *Niban1* [2], *Thrsp* [0]

Combinations of 2 and 3 genes with top probabilities. 0 = no change; 2 = high expression. Data generated from GSE141593 (n = 5 wildtype and PiZ mice).

## Data Availability

The original contributions presented in this study are included in the article/Supplementary Material. Further inquiries can be directed to the corresponding authors.

## Supplementary Materials

The following supporting information can be downloaded at: https://www.mdpi.com/article/10.3390/nu17111913/s1. Supplementary Material Figure S1: Effect of TPEN and zinc treatment on LDH leakage; Figure S2: GeNIe Simulations.

## Abbreviations

The following abbreviations are used in this manuscript:

UPR	Unfolded protein response
MB	Markov blanket
ER	Endoplasmic reticulum
ROS	Reactive oxygen species
BN	Bayesian network
ERAD	ER-associated degradation
AAT	Alpha-1 antitrypsin
AATD	Alpha-1 antitrypsin
ATZ	Alpha-1 antitrypsin mutant Z
PiZ	Alpha-1 antitrypsin mutant Z
AI	Artificial intelligence
