# Supplementary material for: Effect of Alpha-1 Antitrypsin Deficiency on Zinc Homeostasis Gene Regulation and Interaction with Endoplasmic Reticulum Stress Response-Associated Genes
Source: Nutrients. 2025 Jun 2;17(11):1913. doi: 10.3390/nu17111913 (PMC12157045; doi:10.3390/nu17111913)
Supplement: Supplementary file 1 [file nutrients-17-01913-s001.zip › nutrients-3648685-supplementary.pdf]

Supplementary Materials:

The following supporting information can be downloaded at: https://www.mdpi.com/article/doi/s1,

Figure S1. Effect of TPEN and zinc treatment on LDH leakage

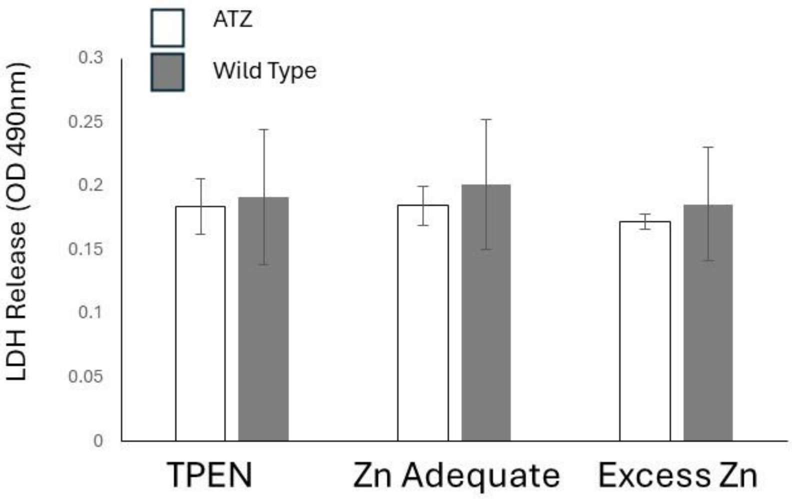

Cells were treated with TPEN (5 μM), Zn Adequate (vehicle alone (DMSO)), or Excess Zn (vehicle plus 40 μM of zinc) for 24 h. Values are averages ± 1 S.D of three independent experiments ±1 S.D. Values were not significantly different ($p > 0.05$) according to One-Way ANOVA.

Figure S2. GeNIe Simulations.

A. 100% PiZ mutation.

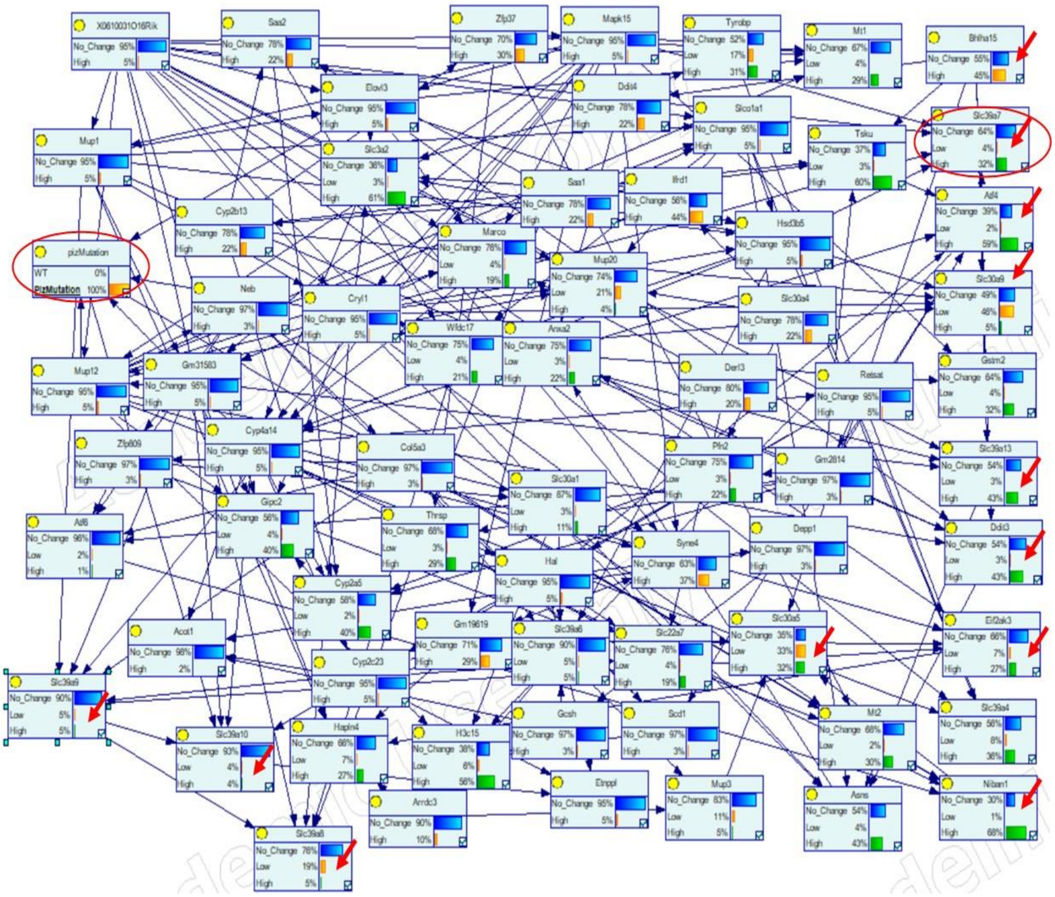

Legend of conditions in bar charts: wildtype (WT) and PiZ (pizMutation).

B. 100% PiZ mutation and 100% high expression of Slc39a7.

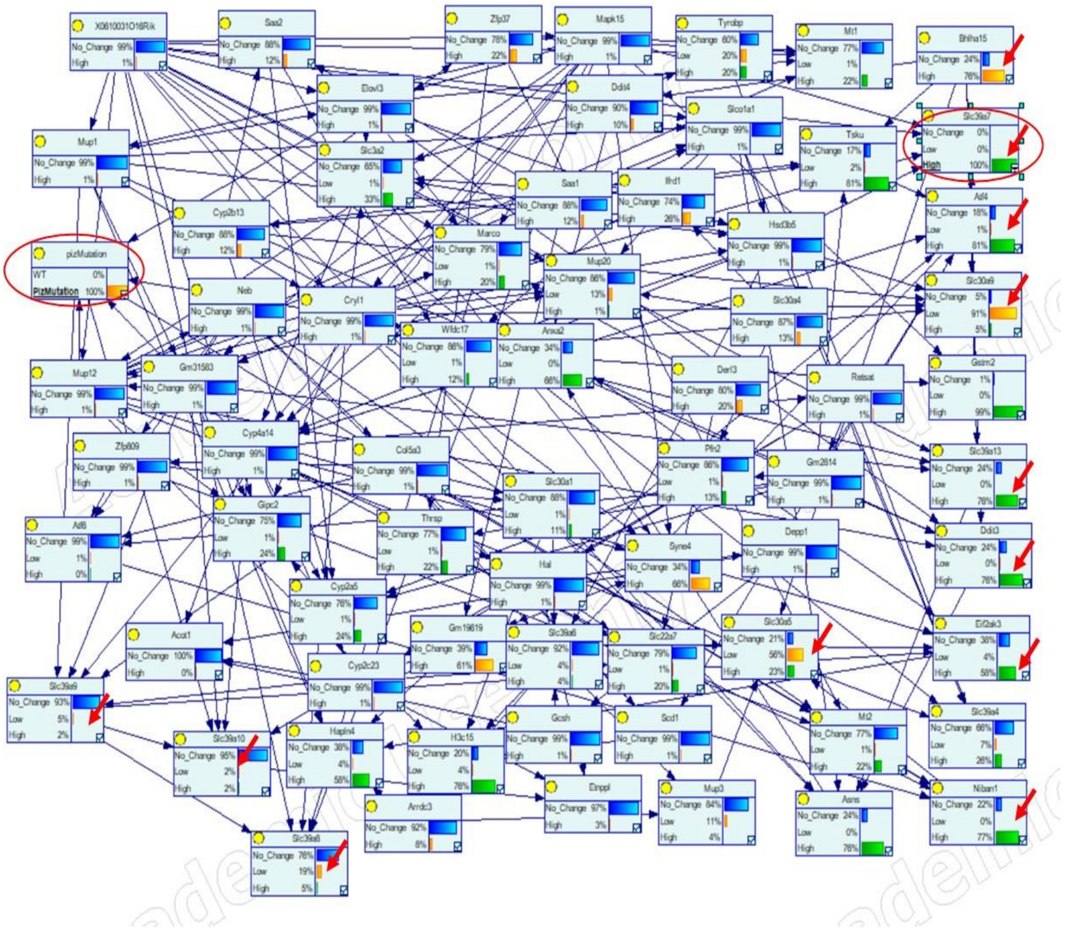

Legend of conditions in bar charts: wildtype (WT) and PiZ (pizMutation).

C. 100% wildtype and 100% high expression of Slc39a7.

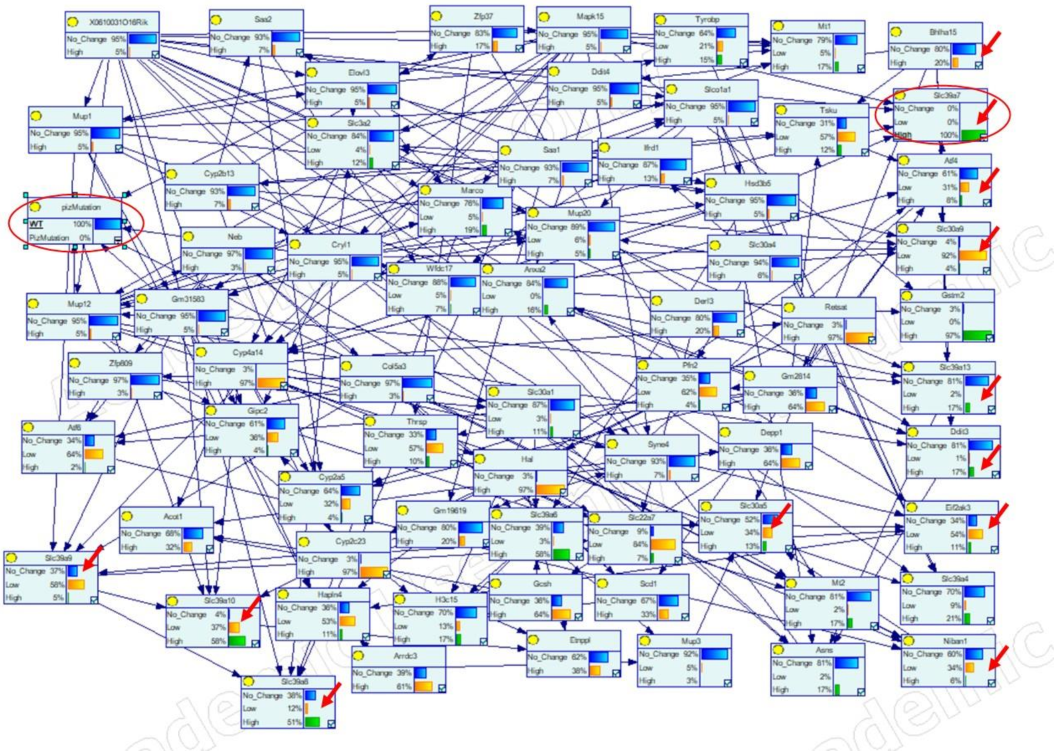

Legend of conditions in bar charts: wildtype (WT) and PiZ (pizMutation).
